# IR Studies of Ethoxy Groups on CeO_2_

**DOI:** 10.3390/molecules28031251

**Published:** 2023-01-27

**Authors:** Jerzy Podobiński, Małgorzata Zimowska, Michał Śliwa, Jerzy Datka

**Affiliations:** Jerzy Haber Institute of Catalysis and Surface Chemistry, Polish Academy of Sciences, Niezapominajek 8, 30-239 Krakow, Poland

**Keywords:** CeO_2_, IR spectroscopy, ethoxy groups, acetate ions

## Abstract

The reaction of ethanol with a surface of CeO_2_ was studied using IR spectroscopy. In some experiments, CeO_2_ was pretreated in a vacuum at 820 K which caused a partial reduction. In other experiments, CeO_2_ was reduced with hydrogen at 770 K. We also used CeO_2_ oxidized by oxygen treatment at 670 K. At low coverages, ethoxy groups and new surface OH groups were formed and water was not produced. On the other hand, at higher loading surfaces, Ce-OH was consumed and ethoxy groups and water were formed. Three kinds of ethoxyls were found on CeO_2_: monodentate, bidentate, and tridentate ones. They were characterized by various frequencies of symmetrical, asymmetrical, and combinational bands of C-C-O units. The reduction of CeO_2_ increased the contribution of tridentate ethoxyls and the oxidation increased the contribution of monodentate ones. At higher temperatures, ethoxy groups were oxidized to acetate ions with the formation of new surface OH groups. Monodentate ethoxyls were the most reactive and tridentate ones were the least reactive during oxidation. The amounts of acetate species were the highest for the oxidized CeO_2_.

## 1. Introduction

Hydrogen may be considered as a very promising fuel, so the production of hydrogen is an important challenge of technology and science. One of the most important methods of hydrogen production is the steam reforming of alcohols. Most studies have been conducted with two alcohols: methanol and ethanol. The advantage of methanol is its easy and efficient production from syngas sources that can be derived from the steam reforming of hydrocarbons or the gasification of coal. Ethanol, on the other hand, is receiving interest as a bio-fuel produced from the fermentation of corn and other renewable resources [1,2,3,4]. 

Most probably, the most important catalytic systems for hydrogen production from alcohols are M/ZnO (M = Cu, Pd) and M/CeO_2_ systems typically promoted by Pt, Pd, or other metals [5,6,7,8,9,10,11,12,13,14,15].

The present study concerns the adsorption and transformations of ethanol on CeO_2_. CeO_2_ has surface centered fluorite type structure which consists of cubic close-packed array of Ce^4+^ cations with all tetrahedral holes occupied by oxygen [16,17]. Surface Ce^4+^ can be reduced to Ce^3+^ in reductive conditions and CeO_2−x_ (0 < x < 0.5) is formed with oxygen vacancies. This makes the reversible addition and abstraction of oxygen possible. The heating of CeO_2_ above 921 K produces CeO_2−y_ (0 < y < 0.18) [18]. In such a phase, the cation sublattice is unchanged and oxygen vacancies are formed. The ease of the reversible abstraction and addition of oxygen means that CeO_2_ may play the role of an oxygen storage system in numerous catalytic reactions. 

One of the catalytic systems in which CeO_2_ is involved is a three-way converter [19,20,21,22] which make a significant contribution to reducing emission levels by converting hydrocarbons, CO, and NO_x_ into nontoxic compounds. The oxidative properties of CeO_2_ and CeO_2_-containing materials were used for the oxidation of some organic compounds [23,24,25]. As mentioned above, another catalytic process based on CeO_2_ is the steam reforming of alcohols and hydrogen production. A lot of the studies on the transformations of methanol and ethanol on CeO_2_ were conducted by Idriss et al. [17,26,27,28,29], Lamotte [30], Binet [31], and Badri [32], as well as other authors [33]. Idriss and collaborators completed complex studies on ethanol transformations on CeO_2_ and CeO_2_ with metals (Pt, Pd, Rh, Au) using IR, TPD, and XPS methods, as well as in catalytic experiments. These authors reported the formation of mono- and bidentate ethoxy groups characterized by 1107 and 1057 cm^−1^ IR bands on the (111) and (310) surfaces of CeO_2_. Ethoxy groups on CeO_2_ were already oxidized to acetate ions at room temperature and to carbonate species above 470 K. The presence of noble metals caused the lowering of the IR frequencies of both monodentate and bidentate ethoxyls, which was interpreted as an increase in the strength of the interaction of ethoxyls with the surface. The addition of Pd changed also the process of oxidation of ethoxy species and ethanol. Higher temperatures were needed to obtain acetate ions, and acetaldehyde was produced. Acetaldehyde was not formed without Pd. Ethoxy groups were also produced on H_2_-reduced CeO_2_, but they were not oxidized at low temperatures. 

Lamotte [30], Binet [31], and Badri [32] completed extensive IR studies on methoxy groups on CeO_2_ as well as the properties of OH groups on unreduced, reduced, and oxidized CeO_2_. The reaction of methoxy groups with CO_2_ producing methyl carbonate was also studied. These authors revealed that fully oxidized CeO_2_ practically did not contain any surface hydroxyls. H_2_-treated CeO_2_ showed four kinds of hydroxyls denoted as OH I (IR band 3730 cm^−1^), two kinds of OH II (OH II A and OH II B—IR bands at 3660 and 3650 cm^−1^, respectively), and OH III (IR band 3600 cm^−1^). The proportion of the amounts of these hydroxyls varied upon hydrogen treatment and upon evacuation. The reaction of methanol with CeO_2_ produced methoxy groups: monodentate, two kinds of bidentate species, and tridentate, characterized by IR bands at 1106, 1062, 1042, and 1015 cm^−1^, respectively. The reduction of CeO_2_ converted monodentate methoxyls into tridentate ones. This process was found to be reversible in the presence of oxygen. Only monodentate methoxyls could react with CO_2_ forming methyl carbonate. Badri et al. [26] also completed quantitative IR studies on methanol adsorption on CeO_2_. The extinction coefficients of the C-O band and the concentration of methoxy groups were determined. 

In this study, using IR spectroscopy, we followed the reaction of ethanol with CeO_2_ surface sites and especially the formation, speciation, and oxidation of ethoxy groups on unreduced, reduced and oxidized CeO_2_. As mentioned above, Lamotte [30], Binet [31], and Badri [32] completed complex IR studies on the interaction of methanol on CeO_2_; however, there was a deficiency of complex IR studies on the ethanol reaction on ceria. Even though Idriss et al. [17,26,29], as well as Jacobs et al. [5], conducted an extensive study on the ethanol transformations on CeO_2_, including IR experiments, there was a lack of data concerning the reactions of ethanol with surface OH groups and the speciation of ethoxyl groups. 

The IR studies of ethanol reactions are a more difficult task than those of methanol transformations because the IR spectrum of ethoxyl groups is more complicated than that of methoxyl groups. While the vibration of C-O fragments in methoxy groups gives only one IR band around 1000 cm^−1^, the vibration of C-C-O entity in ethoxyls gives two bands of symmetric and asymmetric vibration around 900 and 1050 cm^−1^, respectively. Moreover, a third band in this region (at ca. 1100 cm^−1^) is present. This last band was interpreted [34] as the combination of two vibrations: the deformation (δ) of M-O-C and rocking (r) of a CH_3_ group: δM-O-C + r║CH_3_.

## 2. Results and Discussion

### 2.1. Effect of Calcination Temperature

**Figure 1:**
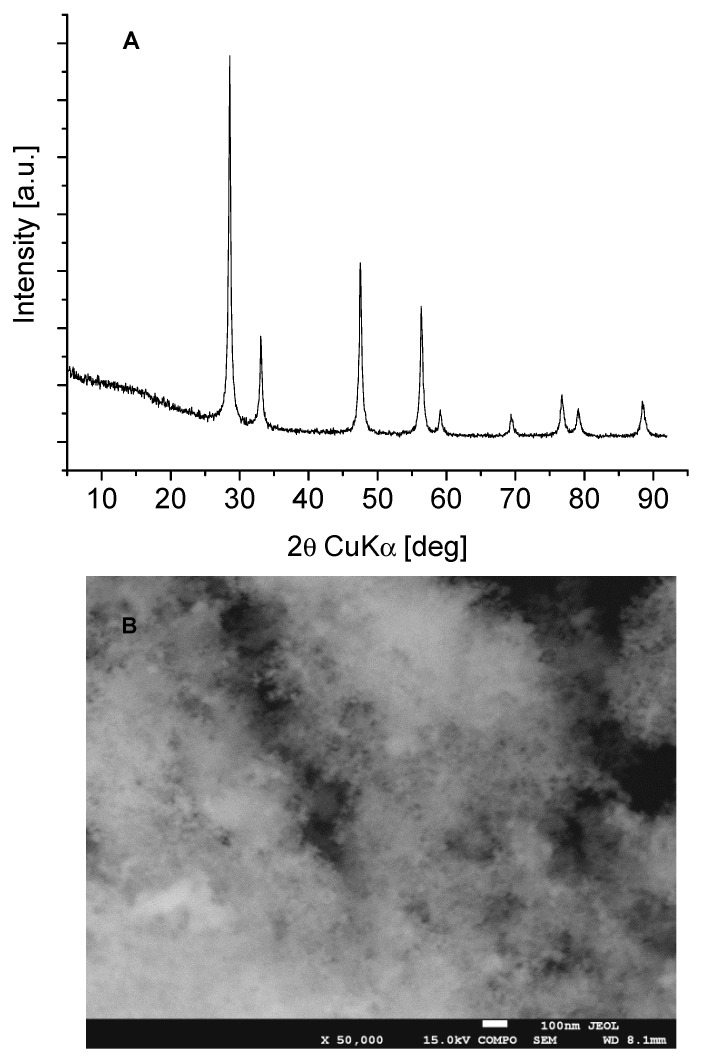
(**A**)—XRD patterns and (**B**)—SEM image of CeO_2_ sample.

The XRD pattern of our CeO_2_ is presented in Figure 1A and the morphology is given in Figure 1B. The diffractogram displays reflections due to the presence of a well-ordered cubic CeO_2_ phase of nanocrystalline character, crystalized in the Fm-3m symmetry [ICDD PDF-4+ 2015 01-075-9470]. SEM analysis reveals that the sample is composed of fine crystallites, with sizes ranging from 18–26 nm (well visible in Figure 1B) which is in line with our XRD calculation of average crystallite size. The surface area determined by N_2_ adsorption was 63 m^2^/g. 

**Figure 2:**
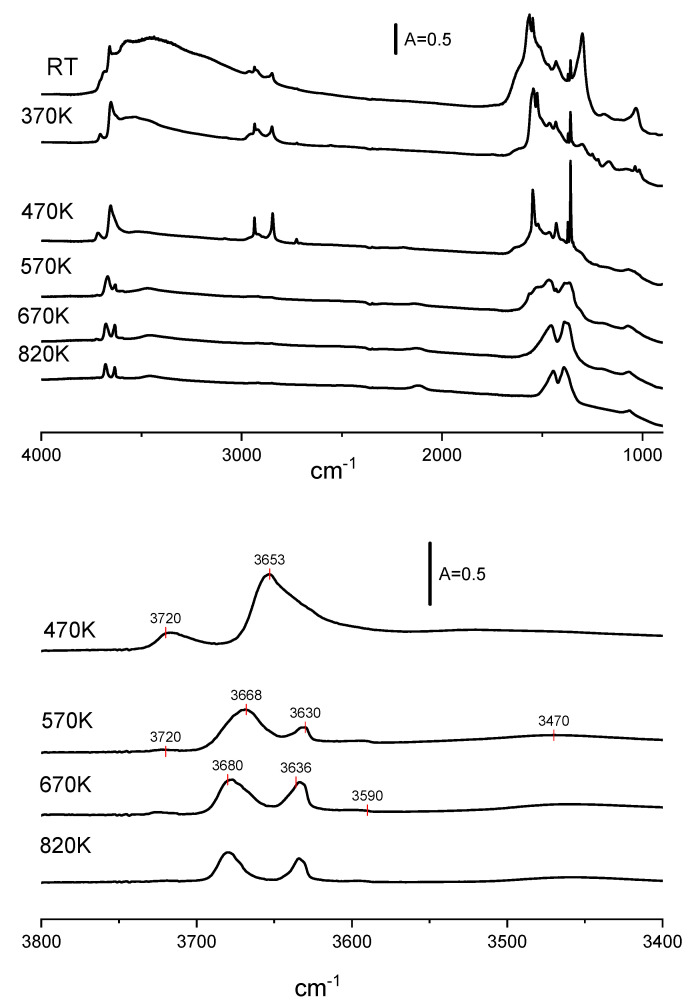
The spectra of OH groups on CeO_2_ activated in a vacuum at various temperatures.

**Figure 3:**
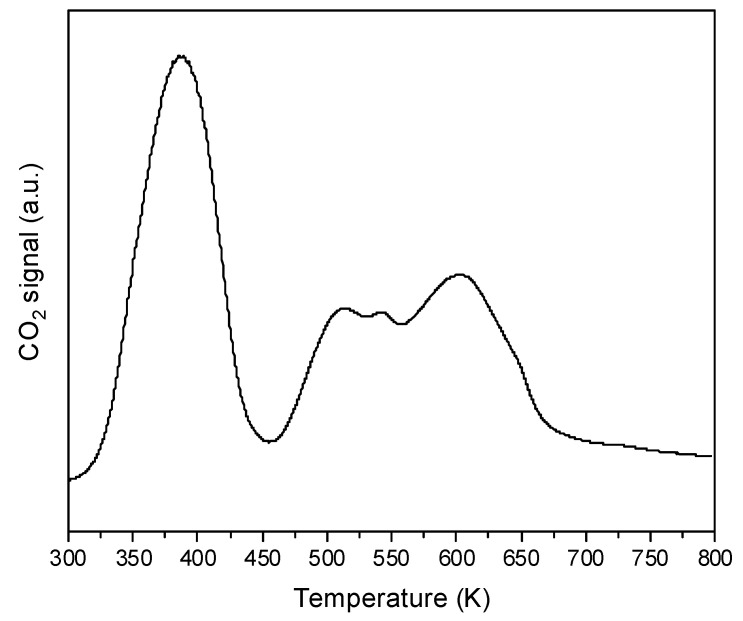
TPD diagram of CO_2_ desorption from CeO_2_.

It is well known that strongly basic CeO_2_ adsorbs atmospheric CO_2_ forming carbonate species. In order to find the optimal conditions of pretreatment, CeO_2_ was calcined in a vacuum at 370, 470, 570, 670, and 820 K. The spectra of such treated CeO_2_ are presented in Figure 2. The calcination above 570 K causes the disappearance of some bands in the region of 1400–1700 cm^−1^ due to the decomposition of carbonates. The decomposition of carbonates was also evidenced in TPD experiments which showed the desorption of CO_2_ during heating (Figure 3). Heating above 570 K causes only small changes in the spectra. In most of the further experiments, CeO_2_ was pre-treated at 570 K in a vacuum.

The spectra of OH groups also change upon evacuation at high temperatures. Two distinct bands are present upon evacuation at 470 K. According to Jacobs et al. [12], they may be assigned to OH (I) (3720 cm^−1^) and to OH (II) (3653 cm^−1^). The last band is broad and most probably it is composed of several submaxima. At a temperature of 570 K and higher, the OH (II) band splits as well as the OH (II A) 3668 cm^−1^ and OH (II B 3630 cm^−1^) bands. A weak OH (III) band at 3590 cm^−1^ is also present. The analysis of the OH (II A) band shows that it is composed of two submaxima around 3680 and 3668 cm^−1^. The hydroxyls at 3668 cm^−1^ are more prone to dihydroxylation and they disappear upon calcination at 670 K. Two distinct bands of OH (IIA) and OH (IIB) at 3680 and 3636 cm^−1^, respectively, are clearly seen upon activation at 820 K, together with weak bands of OH (I) and OH (III) at 3720 and 3590 cm^−1^. The broad and very weak band around 3470 cm^−1^ was attributed to an oxyhydroxy species [31,35].

The spectra of CeO_2_ calcined at 570 K and above show a band at 2126 cm^−1^. The interpretation of the band was discussed by Binet et al. [31]. These authors proposed the electronic origin of this band, either an electronic transition from donor levels located near the conduction band, such as Ce^3+^ or oxygen vacancies [36], or the forbidden ^2^F_5/2_ → ^2^F_7 /2_ electronic transition of Ce^3+^ located at the subsurface or bulk defective lattice sites [37].

### 2.2. Reaction of Ethanol with CeO_2_

**Figure 4:**
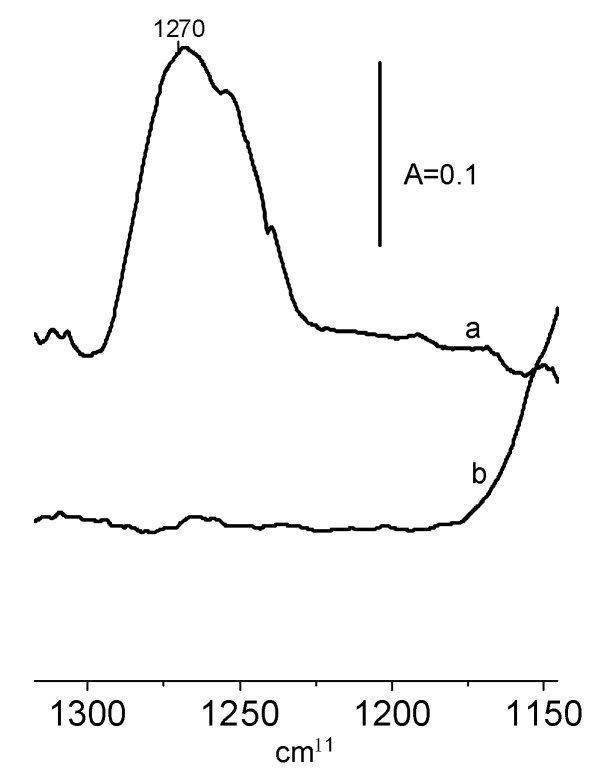
The spectra recorded upon the adsorption of ethanol on CeO_2_ at RT (a), and upon the evacuation at 370 K (b).

The interaction of ethanol with the surface of CeO_2_ is chemisorption. The reaction of ethanol on the CeO_2_ surface produces ethoxy groups. In order to follow the properties and reactivity of ethoxy groups, the doses of ethanol were adsorbed, and subsequently, non-reacted ethanol was removed by evacuation at 370 K. The results presented in Figure 4 evidence that the evacuation at 370 K was sufficient to remove unreacted ethanol—the δ OH band of the deformation vibrations of molecular ethanol at 1270 cm^−1^ disappeared upon the evacuation at this temperature. 

**Figure 5:**
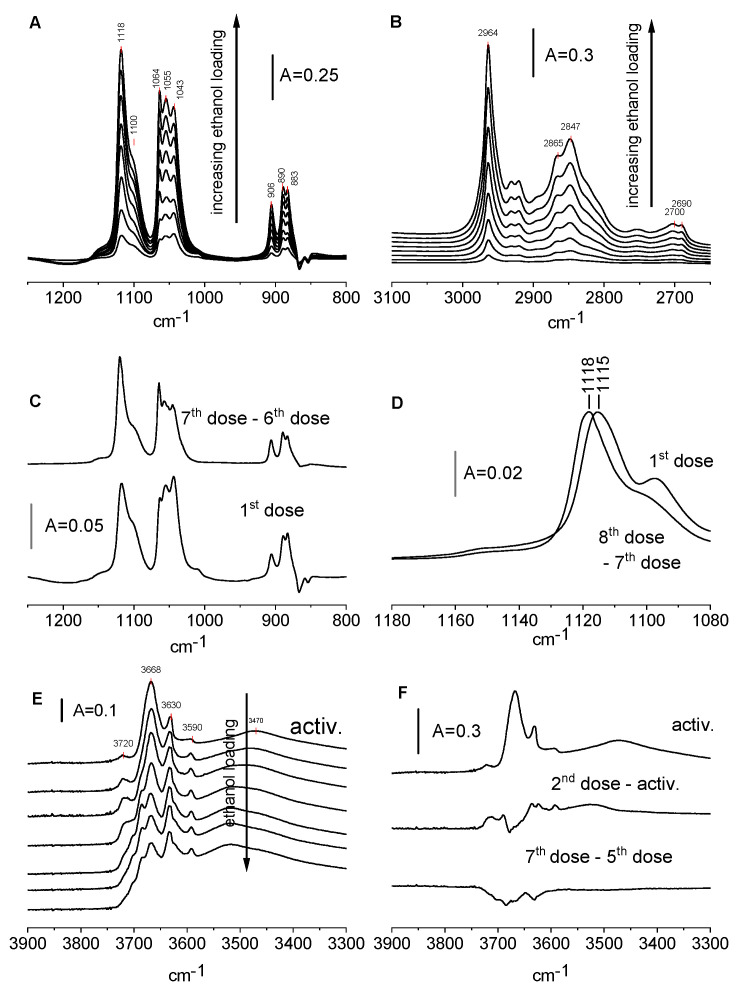
(**A**,**B**)—The spectra of ethoxy groups on CeO_2_ formed upon the adsorption of ethanol. (**C**,**D**)—The spectra of ethoxy groups on CeO_2_ formed by the adsorption of the 1st dose of ethanol and difference spectra (8th dose minus 7th dose). (**E**)—The spectra of OH groups on CeO_2_ and spectra recorded upon the adsorption of ethanol. (**F**)—The spectrum of OH groups on CeO_2_ and difference spectra: second dose minus activated sample and seventh dose minus fifth dose.

The spectra recorded upon the adsorption of several doses of ethanol on CeO_2_ activated at 570 K are presented in Figure 5A,B. The bands at the regions 850–950 cm^−1^ and 1000–1080 cm^−1^ are assigned to the symmetric and asymmetric vibrations of C-C-O fragments, respectively. The bands in the 1080–1150 cm^−1^ region may be assigned to combination vibrations δ_M-O-C_ + r_║CH3_ [28]. In each of these regions, three narrow maxima are seen. 

They are clearly seen for the symmetric and asymmetric vibrations of C-C-O and it is probable that the broad shoulder around 950–1100 cm^−1^ is composed of two submaxima. By analogy with the spectra of the methoxy groups on CeO_2_ [32], we assign the bands at 906, 1064, and 1118 cm^−1^ bands to monodentate, the bands at 890 and 1055 cm^−1^ to bidentate, and the bands at 883 and 1043 cm^−1^ to tridentate ethoxyls. We suppose that the broad shoulder at 1090–1100 cm^−1^ is a superposition of combinational bands of bi- and tridentate ethoxyls. In the region of C-H vibrations, the bands of CH_2_ (2847 cm^−1^), and CH_3_ asym. (2964 cm^−1^). The frequencies of the bands are listed in Table 1, Table 2 and Table 3. The maxima 2690–2700 cm^−1^ are the result of the Fermi resonance of the C-H deformation vibration. 

Figure 5C shows the spectra recorded upon the adsorption of the first dose of ethanol adsorbed on CeO_2_ and the difference spectra: the difference between the last dose and before the last dose of ethanol. Comparing these spectra suggests that tridentate ethoxyls are formed in the first order at lower coverage, whereas monodentate species are formed at the second order. The results presented in Figure 5D concern a combination band of monodentate ethoxyls. This band shifts from 1115 to 1118 cm^−1^ with an increase in the loading. It suggests the presence of several kinds of Ce sites bonding the monodentate ethoxy groups with various frequencies of combination bands.

The spectra of Ce-OH groups upon the reaction with ethanol are presented in Figure 5E and the difference spectra are in Figure 5F.

According to the data presented in Figure 5F, the adsorption of the first doses of ethanol resulted in the formation of new hydroxyl groups. It may be supposed that the following reaction takes place:

CeOCe + HOC_2_H_5_ = Ce-OH + Ce-O-C_2_H_5_ (1)

The adsorption of further doses of ethanol consumes Ce-OH groups according to the scheme:

Ce-OH + HOC_2_H_5_ = Ce-OC_2_H_5_ + H_2_O (2)

**Figure 6:**
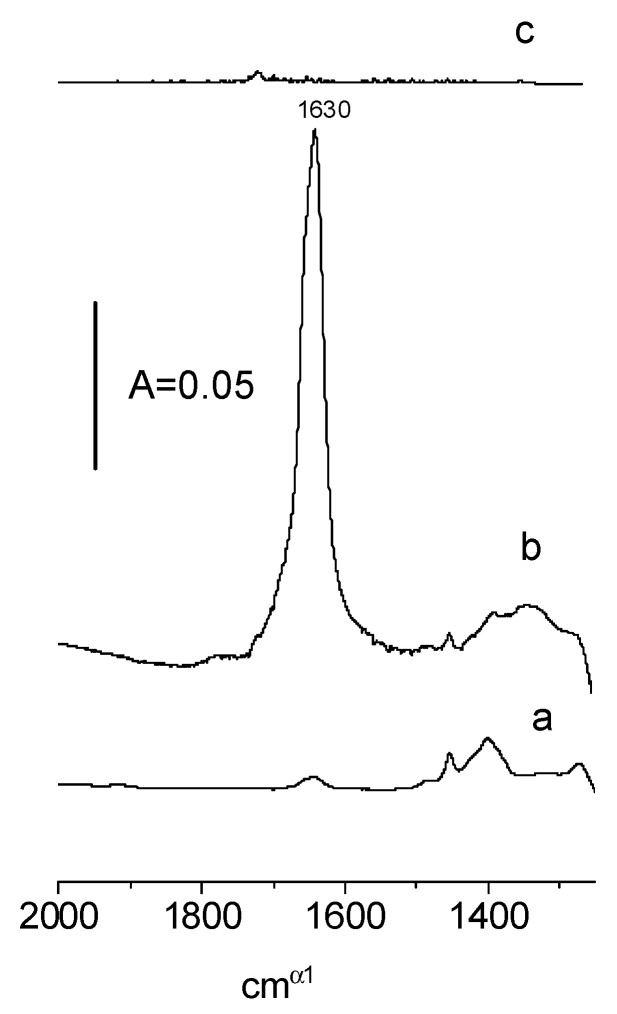
The IR spectra of the products of the reaction of the 1st (a) and 8th dose (b) of ethanol on CeO_2_, as well as the products of the oxidation of ethoxyl groups (c). These products were adsorbed on zeolite NaY.

In order to confirm these hypothetical mechanisms, we conducted experiments, the goal of which was to test if water was definitely formed. In the first experiment, the products of the reaction of the first dose of ethanol were desorbed into the cold trap, and subsequently, they were adsorbed on an activated zeolite disc. A similar procedure was applied in the case of adsorption of the 8th dose. The results are presented in Figure 6. At a low loading of ethanol (spectrum a), water was practically not formed (the band deformation of H_2_O at 1630 cm^−1^ was very small) which agrees with the fact that new hydroxyls were formed and confirms mechanism 1. On the other hand, the 1630 cm^−1^ band was present at higher loadings of ethanol (spectrum b) which agrees with the fact that surface hydroxyls were consumed forming water and confirmed mechanism 2.

### 2.3. Effect of Reduction of CeO_2_ on the Formation of Ethoxy Groups

According to [18], the calcination of CeO_2_ causes the loss of oxygen and CeO_2−y_ (0 < y < 0.18) is formed. In our study, the CeO_2_ was calcined in a vacuum at 820 K, i.e., we obtained a partially reduced sample. We studied also CeO_2_ treated with hydrogen at 770 K for 1 h. After evacuation, the sample was treated with hydrogen at 770 K once more and finally was evacuated at 670 K. Such a sample was reduced to a bigger extent than the one which was calcined at 820 K only. 

**Figure 7:**
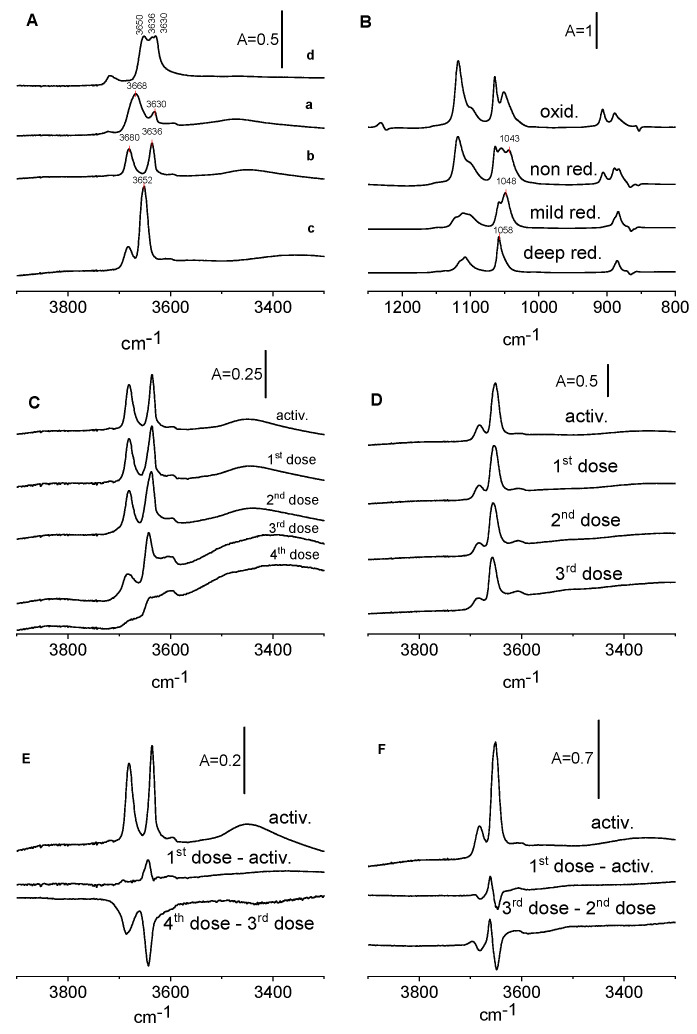
(**A**)—IR spectra of OH groups on non-reduced CeO_2_ (activated at 570 K) (a), mildly reduced (activated at 820 K) (b), deeply reduced by hydrogen at 770 K (c), and oxidized by oxygen at 670 K (d). (**B**)—ethoxy groups formed on non-reduced CeO_2_, mildly reduced, deeply reduced, and oxidized. (**C**,**D**)—OH groups on CeO_2_ mildly reduced (**C**) and deeply reduced (**D**) upon the adsorption of ethanol. (**E**)—The spectrum of OH groups on mildly reduced CeO_2_ and difference spectra: first dose minus activated sample as well as 4th dose minus 3rd dose of ethanol. (**F**)—The spectrum of OH groups on deeply reduced CeO_2_ and difference spectra: first dose minus activated sample as well as 3rd dose minus 2nd dose of ethanol.

The spectra of OH groups on the surface of reduced CeO_2_ are presented in Figure 7A. Activation at 820 K (spectrum b) causes the dehydroxylation and loss of 3668 cm^−1^ hydroxyls. Reduction with hydrogen (spectrum c) does not change the hydroxyls vibrating at 3680 cm^−1^ (OH II A) but increases the concentration of OH II B (3636 cm^−1^). This may be the result of the reduction of CeO_2_ and the formation of new hydroxyls. The band of OH II B shifts from 3631 to 3636 cm^−1^. 

Ethanol was subsequently adsorbed on reduced samples of CeO_2_. The spectra of ethoxy groups are presented in Figure 7B and the spectra of OH groups are given in Figure 7C,D. According to the data presented in Figure 7B, the mild reduction by calcination at 820 K caused the distinct decrease in the amount of monodentate ethoxy groups, whereas on the deeply reduced CeO_2_ surface, the adsorption of ethanol lead to the formation of only tridentate ethoxyls. A more significant reduction by hydrogen treatment also caused the loss of most of the bidentate ethoxyls, with little more than tridentate ethoxyls remaining. A similar situation was observed by Binet et al. [31] for methoxy groups. These authors reported that the reduction of CeO_2_ converted monodentate methoxyls into tridentate ones. This process was found to be reversible in the presence of oxygen [31].

Figure 7C,D present the spectra of OH groups in reduced CeO_2_ before and after the reaction with ethanol. The difference spectra are shown as well (Figure 7E,F). As mentioned, CeO_2_ that was moderately reduced by activation at 820 K shows distinct OH bands of OH(IIA) and OH(2B) at 3680 and 3636 cm^−1^ and smaller bands of OH(1) and OH(III). The band of OH(I) is smaller than that before the reduction. It is possible that the distinct decrease in the amount of monodentate ethoxy groups may be related to the elimination of OH (I) by dehydroxylation. 

The analysis of difference spectra (Figure 7E,F) suggests that in CeO_2_ which is mildly reduced by the activation (similarly to non-reduced CeO_2_) of the first doses of ethanol at 820 K, new hydroxyls are produced, and the next doses consume OH. For CeO_2_ deeply reduced by hydrogen all the doses of ethanol consume some hydroxyls and produce new ones, suggesting that both mechanisms 1 and 2 overlap. 

### 2.4. Effect of Oxidation of CeO_2_ on the Formation of Ethoxy Groups

The oxidation of CeO_2_ was realized by the treatment of the sample with oxygen at 670 K, which was previously activated in a vacuum at 570 K. After oxygen treatment, CeO_2_ was evacuated at 570 K. 

The oxidation changed the OH groups (Figure 7A). The OH (II A) band at 3680 cm^−1^ disappeared and the band at 3668 cm^−1^ shifted to lower frequencies, increasing distinctly, and showed three submaxima at 3630, 3636, and 3650 cm^−1^. The amount of OH (III) increased significantly. 

The spectra of ethoxy groups in non-oxidized and oxidized CeO_2_ are presented in Figure 7B. The oxidation of CeO_2_ causes an increase in the amount of monodentate and a decrease in the amount of tridentate ethoxyls. 

The analysis of the spectra of OH groups evidenced (spectra not shown) that at lower coverages, some hydroxyls are formed, whereas at higher coverages, hydroxyls are consumed. 

Summing up, it can be said that monodentate ethoxyls predominate for oxidized CeO_2_ and tridentate ones for the reduced samples. 

### 2.5. Frequencies of Ethoxyl Groups

The results obtained in this study enable us to compare the stretching frequencies of mono-, bi-, and tridentate ethoxy groups The frequencies of C-C-O fragments of these species taken from Figure 5A are presented in Table 1. All the frequencies of stretching vibrations are the highest for monodentate ethoxyls and the lowest for tridentate species. This is because the higher the coordination number, the lower the strength of the bonds.

It was also possible to follow the effect of oxidation and reduction of CeO_2_ on the stretching frequencies of C-C-O fragments in ethoxyls. Figure 7B presents the spectra of ethoxy groups formed on non-reduced, reduced, and oxidized CeO_2_, and the band frequencies are given in Table 2. According to these data, the reduction of CeO_2_ caused the increase in stretching frequencies of asymmetric C-C-O units. Similar effects were reported by Binet [31] for methoxy groups. On the other hand, the oxidation of CeO_2_ causes the decrease in the band frequencies.

**Figure 8:**
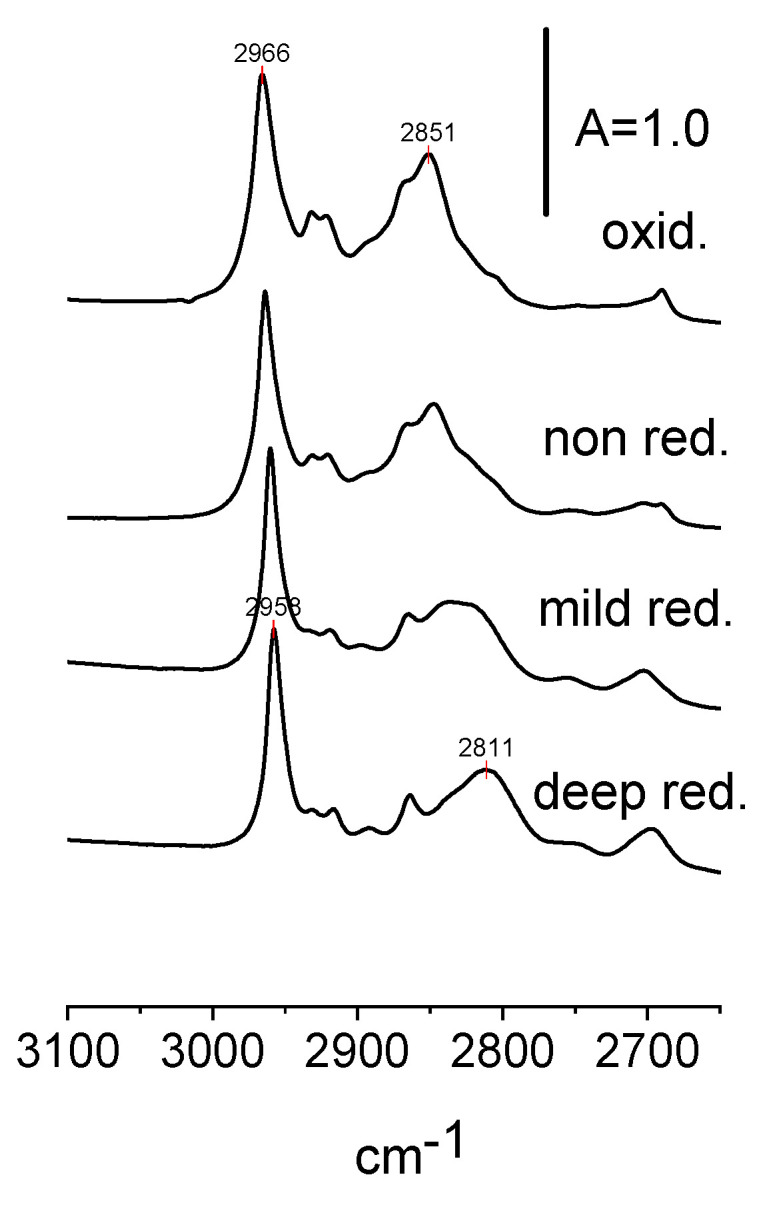
The bands of CH_2_ and CH_3_ groups in ethoxyls formed on the surface of non-reduced, reduced and oxidized CeO_2_.

The spectra of the CH stretchings of ethoxy groups in non-reduced, reduced, and oxidized CeO_2_ are presented in Figure 8 and the frequencies of CH_2_ and CH_3_ are given in Table 3. Both frequencies are the lowest for reduced and the highest for oxidized CeO_2_. For CH_2_, the effect is more significant than for CH_2_, because the CH_2_ group is closer to the adsorption site on the CeO_2_ surface. We suppose that oxidation and reduction change the status of Ce ions (oxidation, coordination, and/or environment) on the surface and changes the properties of ethoxy groups. Another effect that affects the C-H frequencies is the multiplicity of the bonding of Ce-O bonds on the properties of ethoxy groups. It affects the frequencies of both C-C-O and C-H stretching. The information on the effect of the multiplicity of Ce-O bonds on the properties of bonds in C-C-O in ethoxy groups is given in Table 1. Both effects influence the C-H stretching (Table 3).

### 2.6. Oxidation of Ethoxy Groups

**Figure 9:**
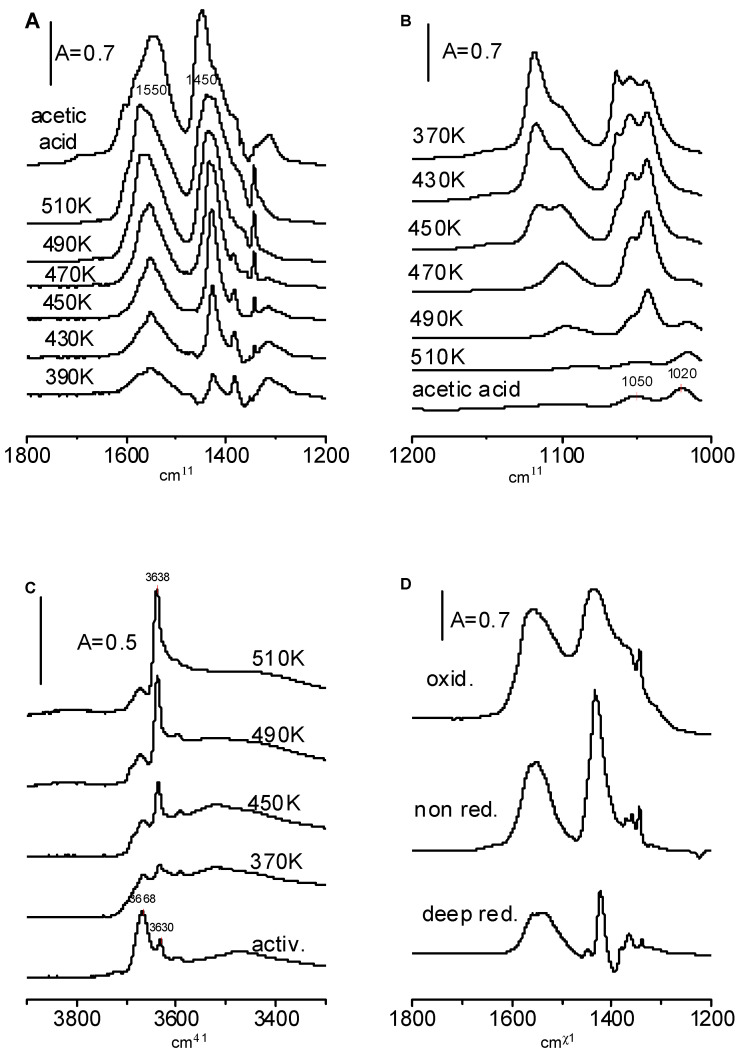
(**A**–**C**)—The spectra recorded upon the formation of ethoxy groups on CeO_2_ and heating. (**D**)—The spectra of acetate ions formed on non-reduced, deeply reduced, and oxidized CeO_2_.

Ethoxy groups on CeO_2_ are oxidized to acetate ions. The maxima characteristic of acetate ions appear at 1450 and 1550 cm^−1^ (symmetric and asymmetric COO^−^ stretching). These maxima are seen in Figure 9A (top spectrum) in which the spectrum of acetic acid adsorbed at room temperature on CeO_2_ is shown. Weak bands at 1050 and 1020 cm^−1^ are also present (Figure 9B, bottom spectrum) 

Acetic species were formed when CeO_2_ was heated with ethoxy species (Figure 9A). The bands of acetic ions increase significantly and the bands of ethoxy groups decrease with the temperature (Figure 9B). The weak band of acetate ions at 1020 cm^−1^ becomes visible at higher temperatures. 

The data presented in Figure 9B evidence that mono-, bi-, and tridentate ethoxyls show various reactivities during oxidation by CeO_2_. The monodentate ethoxyls are completely oxidized at 470 K. Most of the bidentate ethoxyls are completely oxidized at 490 K and tridentate ethoxyls are oxidized at 510 K. 

As mentioned above, the reaction of the first doses of ethanol on the CeO_2_ surface created new hydroxyls, but the next doses consumed hydroxyls. According to the data presented in Figure 9C, the oxidation of ethoxy groups to acetate ions created OH groups. We suppose that the hydrogen which is released in the process C_2_H_5_O-Ce → CH_3_ COO^−^ + Ce^+^ reacts with surface oxygens forming hydroxyls. This hypothesis was additionally supported in the experiment in which the products of the oxidation of ethoxy groups and the formation of acetate ions were “trapped” in the cold trap and, subsequently, these products were adsorbed on activated zeolite NaY. The spectrum shown in Figure 6 (spectrum c) evidences that no water was formed; therefore, the hydrogen that was released was engaged in the formation of new hydroxyl groups.

The heating of ethoxy groups on reduced and oxidized CeO_2_ also produces acetate ions. This is seen in Figure 9D in which the spectra recorded upon the heating of CeO_2_ with ethoxyl groups to 490 K are presented. The amount of acetate ions is the lowest for the reduced CeO_2_ and the highest for oxidized CeO_2_.

## 3. Materials and Methods

Cerium(IV) oxide CeO_2_ nanopowder obtained from Aldrich was used (purity 99,5%). 

X-ray diffraction (XRD) patterns were collected with the X’Pert PRO MPD diffractometer (PANalytical, Almelo, The Netherlands) with CuKα radiation (40 kV, 30 mA) selected by a nickel monochromator in a diffraction beam with a step size 0.05°. The pattern was recorded in the range of 2–92° with the use of a silicon low background sample holder. The crystal size of the oxide was estimated using the Scherrer equation based on the fwhm (full width at half-maximum) measurement of the reflections.

The morphology of the sample was carried out by means of a JEOL JSM–7500F Field Emission Scanning Electron Microscope (JEOL, Akishima, Japan) equipped with a retractable backscattered-electron detector (RBEI) and energy dispersive spectra (EDS) detection system of a characteristic X-ray radiation AZtec Live for EDS system (Oxford Instruments, London, UK).

The specific surface area was determined from the nitrogen adsorption–desorption isotherms obtained at –196 °C using a Quantachrome Nova 2000 apparatus. The specific surface area measurement was based on BET (Brunauer-Emmett-Teller) formalism.

The temperature-programmed desorption experiment (TPD) was carried out in a quartz fixed-bed flow reactor connected online to the mass spectrometer (QMG 220 PRISMA PLUS). Signal *m*/*z* = 44 (CO_2_) was monitored during the TPD. For the analysis, 50 mg of the sample was placed in the reactor. Prior to TPD, the sample was kept at room temperature in a stream of He until line *m*/*z* = 44 was stable. Next, the TPD was performed from RT to 800 K with ∆T = 10 deg/min.

For the IR studies, CeO_2_ was pressed into thin wafers of ca. 150—200 mg. Prior to the IR experiments, wafers were evacuated in situ in an IR cell at 570 K or 820 K for 30 min. In some experiments, CeO_2_ was reduced by hydrogen treatment at 770 K for 1 h. Next, hydrogen was removed by evacuation at 670 K and the reduction procedure was repeated at 770 K. Finally, the wafer was evacuated at 670 K. In other experiments, CeO_2_ was oxidized by oxygen treatment at 670 K followed by evacuation at 570 K. 

The doses of gaseous ethanol (ca. 20 µmol/g) were adsorbed at room temperature and, subsequently, unreacted ethanol was removed by evacuation at 370 K. The IR spectra were recorded at room temperature. The spectra were recorded with a NICOLET 6700 spectrometer (Thermo Scientific, Cambridge, MA, USA) with a spectral resolution of 1 cm^−1^.

## 4. Conclusions

The adsorption of ethanol on CeO_2_ results in the formation of ethoxy groups. At low ethanol loadings, water is not produced and new hydroxyls are formed; at higher coverages, water is formed and hydroxyls are consumed. Monodentate, bidentate, and tridentate ethoxyls are formed. Each of these ethoxyls is characterized by IR bands of the symmetric and asymmetric vibrations of C-C-O units as well as of the combinational band. At lower loadings, tridentate ethoxyls are preferentially formed, and at higher loadings, monodentate. The reduction of CeO_2_ eliminates the sites responsible for the formation of mono- and bidentate ethoxyls; only tridentate are observed in the deeply reduced sample. On the other hand, monodentate ethoxyls dominate on oxidized CeO_2_. The frequencies of asymmetric C-C-O stretching, as well as of CH_3_ and CH_2_, depend both on the multiplicity of the bond of ethoxy groups with the surface and on the oxidation state of the oxide. Ethoxyl groups on CeO_2_ are oxidized to acetate ions. Monodentate ethoxyls are oxidized at lower temperatures, whereas higher temperatures are needed to oxidize tridentate species. The oxidation of ethoxyls does not produce water. Surface hydroxyls are formed.

## Figures and Tables

**Table 1:** The frequencies of monodentate, bidentate, and tridentate ethoxy groups.

	Monodentate	Bidentate	Tridentate
sym. C-C-O	906	890	883
asym. C-C-O	1064	1055	1043
combination	1118	a	a

a—cannot be measured because of band overlapping.

**Table 2:** The frequencies of C-C-O asymmetric bands of ethoxy groups formed of non-reduced, reduced and oxidized CeO_2_.

	Monodentate	Bidentate	Tridentate
oxidized	1064	1051	1040
non reduced	1064	1055	1043
mildly reduced	a	1058	1048
deeply reduced	a	a	1058

a—band absent.

**Table 3:** The frequencies of CH_3_ and CH_2_ groups.

	CH_3_	CH_2_
oxidized	2966	2851
non reduced	2964	2847
mildly reduced	2960	2829
deeply reduced	2958	2811

## Data Availability

Not applicable.

## Sample Availability

Samples of the compounds are not available from the authors.
